# Cost and burden of gastroesophageal reflux disease among patients with persistent symptoms despite proton pump inhibitor therapy: an observational study in France

**DOI:** 10.1186/1471-230X-13-39

**Published:** 2013-02-28

**Authors:** Stanislas Bruley des Varannes, Helena Granstedt Löfman, Maria Karlsson, Peter Wahlqvist, Magnus Ruth, Mary Lou Furstnau, Nicolas Despiégel, Nils-Olov Stålhammar

**Affiliations:** 1 Institut des Maladies de l’Appareil Digestif, University Hospital – CHU Hôtel Dieu, Place A Ricordeau, Nantes Cedex, 44093, France; 2 AstraZeneca R&D, Mölndal, Sweden; 3 OptumInsight, Nanterre, France

**Keywords:** PPIs, GERD, Quality of life, Productivity

## Abstract

**Background:**

Gastrointestinal reflux disease (GERD) is a common disorder that negatively impacts health-related quality of life (HRQL) and work productivity. Many patients have only a partial response to proton pump inhibitor (PPI) therapy and continue to experience GERD symptoms despite optimized treatment. This observational study aimed to provide information on symptoms, HRQL, resource usage, costs and treatment pathways associated with partial response to PPI therapy in French patients with GERD.

**Methods:**

Patients with partial response to PPI therapy, defined as persistent GERD symptoms ≥3 days/week despite optimized treatment with a PPI, were recruited for this 12-month observational study. GERD symptoms, HRQL, work productivity and resource use were assessed by patient surveys. Costs were calculated based on lost work productivity and resource use.

**Results:**

The patient population (n=262; mean age, 54 years; 40% men) carried a significant symptom burden, with 98% of patients having moderate-to-severe GERD symptoms and 65% of patients experiencing daily symptoms at baseline. HRQL and work productivity were significantly impaired, with a greater degree of impairment in patients with higher symptom burden. The mean total cost per patient over the 12-month follow-up period was €5237, of which €4674 (89%) was due to lost work productivity.

**Conclusions:**

Partial response to PPI therapy for GERD is associated with a high symptom burden, significant impairment of HRQL and work productivity, and substantial GERD-related costs.

## Background

Gastroesophageal reflux disease (GERD) is a common disorder, the predominant symptoms of heartburn and/or regurgitation affecting approximately 10–20% of the adult population in the Western world [1]. A national survey showed that the prevalence of GERD in France is at the lower end of this scale, with approximately 10% of the adult population affected by at least one typical symptom such as heartburn and acid regurgitation [2]. The survey also noted that GERD symptoms occurred daily in about 15% of patients, and at least once weekly in 73%, with negative impact on health-related quality of life (HRQL). Such impact was most pronounced in patients with higher symptom frequency, extra-digestive symptoms and endoscopic lesions of greater severity.

Treatment with a proton pump inhibitor (PPI) is generally effective for the treatment of GERD [3]. However, 17–32% of patients have only a partial response and continue to experience GERD symptoms despite optimized therapy with a PPI [4]. Indeed, a study of partial response to PPI treatment in the USA confirmed the burden of disease among such patients [5]. For example, patients experienced an approximate 30% reduction in work productivity, and a 41% reduction in productivity during daily activities, with the degree of productivity and HRQL impairment being positively correlated with the severity and frequency of GERD symptoms despite optimized PPI therapy [5]. Currently, however, there are little data on disease burden, resource use, costs and treatment pathways in this patient population in Europe.

The REMAIN (Partial Response to PPI treatment: the Cost to Society and the Burden to the Patient) France study therefore aimed to provide information on the treatment pathways, resource utilization, costs, symptoms and HRQL in European patients with GERD who were newly identified as partial responders to optimized PPI therapy.

## Methods

### Study design and patients

REMAIN France was a 12-month multicenter, non-interventional, observation study conducted at 50 primary and secondary care sites in France (NCT00842855; AstraZeneca study code: D9120N00013). Participating sites comprised primary care (n=24) and specialist (gastroenterologist) centers (n=26). The study enrolled patients aged 18 years or older with a diagnosis of GERD (established ≥6 months previously) who were newly identified as partial responders to optimized PPI therapy during regular physician visits (pre-arranged appointments were not set up for patients to be screened for enrollment). Partial response was defined pragmatically as GERD symptoms despite optimized PPI therapy, according to the physician’s judgement. In this regard, physicians were asked to record the reason why PPI therapy was considered optimized, including ‘maximum dose’ (physicians were not provided with guidance in terms of what constituted ‘maximum’ dose), ‘tried different brands with no improvement’, ‘improvement by changing PPI unlikely due to symptom pattern’ and ‘other reason’ (multiple responses possible). In addition, patients had to report ≥3 days/week of a burning feeling behind the breastbone (‘heartburn’) of at least moderate intensity and/or ≥3 days/week of an unpleasant movement of material upwards from the stomach (‘regurgitation’) of at least moderate intensity, as determined by the validated Reflux Symptom Questionnaire 7-day recall (RESQ-7) [6].

Patients who had not experienced any GERD symptom improvement during PPI therapy, who had prior surgery of the upper gastrointestinal tract or had any condition that would render the patient unsuitable for inclusion in the study, were excluded.

Written informed consent was obtained from all enrolled patients, who were not remunerated for their participation. Physicians received compensation for their involvement in the study. The study protocol was approved by the Commission Nationale Informatique et Liberté (CNIL).

### Patient-reported outcomes

Patient-reported outcomes were evaluated in terms of GERD-specific symptoms, HRQL and work productivity during follow-up, as described below. Paper-based forms/surveys were used, and considerable steps were taken to ensure their completeness as part of usual study monitoring. Indeed, patient surveys were completed in the physician office, which allowed for reminders to be made if information was incomplete.

### Symptoms

GERD-specific symptoms were evaluated at baseline and at 3, 6, 9 and 12 months’ follow-up using RESQ-7 [6], a patient-reported outcome instrument based on the Reflux Disease Questionnaire [7]. The RESQ-7 is self-administered questionnaire that uses 26 questions to assess the frequency and intensity of GERD symptoms over the past 7 days. Symptoms are rated on a 6-point Likert scale, with higher scores indicating more frequency or intense symptoms. The individual symptom items can be combined into an Overall symptoms domain and four separate domains comprising: Heartburn; Regurgitation; Hoarseness, cough and difficulty swallowing; and the single item Burping.

Depression and anxiety were assessed on the Hospital Anxiety and Depression Scale (HADS) [8] at baseline and 12 months’ follow-up. The HADS is a 14-item self-assessment scale, with each item scored on a four point response category (0–3). The scale produces an anxiety score and a depression score (0–7, no disorder; 8–10, ‘possible’ anxiety or depression; ≥11, ‘probable’ anxiety or depression).

### Health-related quality of life

HRQL was assessed at baseline and at 6 and 12 months’ follow-up using the 36-item Short-Form Health Survey (version 2 acute, SF-36v2) with a 7-day recall period [9] and the European Quality of Life-5 Dimensions (EQ-5D) [10]. SF-36v2 is a generic quality-of-life instrument that assesses physical functioning, bodily functioning and role limitations due to health problems, role limitations due to personal or emotional problems, emotional well-being, social functioning, energy/fatigue, general health problems and perceived change in health. It yields an eight-scale profile of functional health and well-being scores as well as Physical and Mental Component summary scores (0–100), with higher scores indicating a more favorable health state.

The EQ-5D is a health-outcome measure that provides a descriptive profile and an index score for health status. The index score is based on five questions covering aspects of health (mobility, self-care, usual activities, pain/discomfort and anxiety/depression) across three levels (no problems, some/moderate problems and extreme problems). In addition, another measure of health status is assessed on a vertical graduated visual analog scale. The index and VAS scores are both presented as values between 0 and 1, where 0 represents a health state of being dead and 1 represents a health state of being at full health.

### Work productivity

Work productivity was assessed using the Work Productivity and Activity Impairment-GERD (WPAI-GERD) questionnaire [11] at baseline and at 3, 6, 9 and 12 months’ follow-up. The WPAI-GERD consists of six questions, and assesses GERD-related impairment of work productivity and activity over the past 7 days. The questionnaire yields a number of scores, such as: absenteeism (hours of work time missed; employed patients); presenteeism (impairment at work/reduced on-the-job effectiveness; employed patients); and activity impairment because of GERD (employed and non-working patients). Presenteeism and activity impairment outcomes are expressed as percentages, with higher percentages indicating greater impairment and less productivity. The number of work hours lost due to presenteeism is calculated by multiplying the percentage reduced productivity while at work by the number of hours the responder actually worked.

### GERD-related resource utilization and costs

Resource utilization specific to GERD was evaluated in terms of medication use, medical consultations, emergency room visits, hospitalizations and tests and procedures during the study. As part of retrospective data collection, physicians noted which medications considered to be relevant for patient care were prescribed during the 6 months before baseline, along with tests and procedures that were performed.

For the 12-month follow-up phase of the study, patients self-reported on prescribed GERD and over-the-counter (OTC) medications, including treatment duration for each therapy. Such information was used to describe common treatment pathways for the patient population during the course of the study.

Medical consultations, emergency room visits and hospitalizations during the study were reported by physicians and patients by means of case report forms (CRFs) and survey forms, respectively. In cases where the number of primary care physician/specialist visits reported by patients was inconsistent with the number of visits reported by physicians, the maximum number of visits (between patient- and physician-reported information) was used. The justification for taking this approach, when the physician reported a higher number of visits, was that the patient had probably forgotten to report some of the visits. Conversely, the number of visits reported by patients was sometimes higher as the patient could visit other physicians than the study investigator.

Medication costs were derived from the Caisse Nationale d’Assurance Maladie des Travailleurs Salariés (CNAMTS) using basic medicines and pricing information [12]. Costs of non-reimbursed medications were obtained directly from a pharmacist. Prescribed and OTC medications were included in the total direct cost calculation, as this analysis was performed using the societal perspective.

Medical consultation costs were obtained from CNAMTS using conventional tariffs for the public sector [13], and from the Ecosanté France database for the private sector [14]. The unit cost used was a weighted average of the two sectors according to proportions reported in 2006 [15]. Charges for diagnostic procedures were also obtained from the CNAMTS according to the Classification Commune des Actes Médicaux (CCAM).

The cost of productivity loss was calculated using a mean hourly labor cost of €31.97, obtained from the labor costs section in the Eurostat website [16]. The number of hours absent from work and number of hours lost due to reduced work productivity over the past 7 days were calculated using the WPAI-GERD results. The number of hours collected at baseline was applied for the 6-month period prior to baseline. Similarly, the number of hours at 3 months was applied for the period between baseline and 3 months, the number of hours at 6 months was applied for the period between 3 months and 6 months, the number of hours at 9 months was applied for the period between 6 months and 9 months, and the number of hours at 12 months was applied for the period between 9 months and 12 months. Productivity loss was adjusted to reflect the total study population when calculating productivity costs per patient.

#### Statistical analyses

All statistical analyses were descriptive, due to the objectives of the study. Consequently, there are no hypotheses to test with statistical methods to predetermine a required sample size. The protocol specified a target enrollment of 275 patients with GERD and partial response to optimized PPI treatment. Assuming an attrition rate of 30% over the course of the study, the final sample size would be approximately 200 patients for analysis. This sample size was pragmatic and deemed sufficient given the descriptive objectives of the study. 95% confidence intervals (CIs) were used to visualize the variability of estimates, and were based on normal distribution approximations. No adjustments were made to account for missing data.

## Results

### Patient characteristics

**Figure 1:**
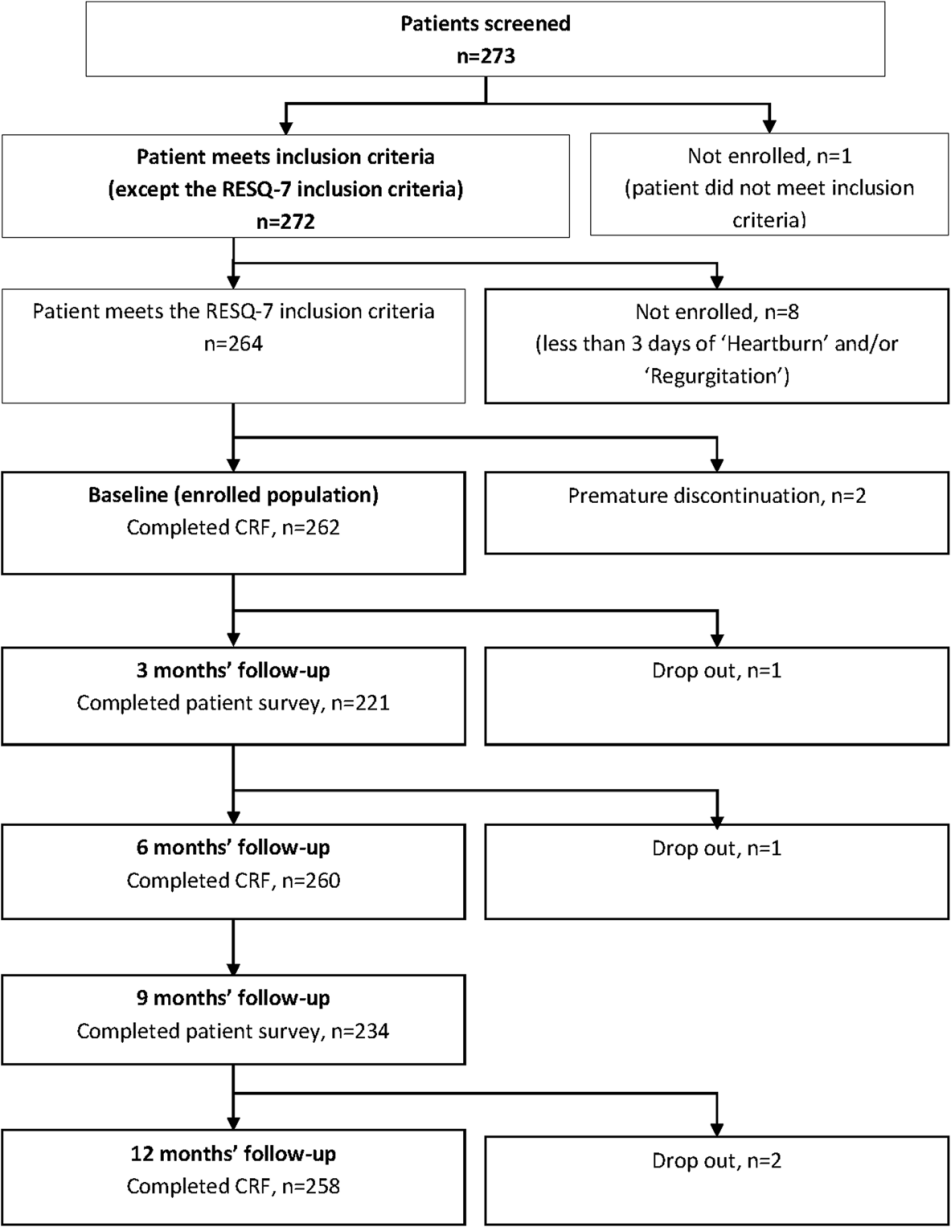
Flow of patients through the REMAIN France study and follow-up process.

A total of 273 patients were screened for inclusion in the study, of which 264 patients met the inclusion criteria. At the baseline evaluation, physicians completed a CRF for 262 patients, and 260 patients completed the patient survey. Four patients dropped out over the course of the study, with CRFs and patient surveys completed for 258 patients and 226 patients, respectively, at the 12-month follow-up visit. The flow of patients through the study is depicted in Figure 1.

The mean age of the study population was 54 years, 40% of patients were men and the mean body mass index was 26.6 kg/m^2^. A total of 49% of patients were employed (2% were on sick leave), 15% were unemployed and the remaining 36% of patients were retired. On the HADS scale, 21.5% of patients were classified as having probable anxiety and 13% were classified as having probable depression.

The reasons for considering PPI therapy to be optimized were recorded by physicians (multiple response possible); the ‘maximum dose’ had been reached in 86% of patients, 46% of patients had tried a different brand with no improvement, improvement by changing PPI was considered unlikely due to symptom pattern in 29% and PPI therapy was considered optimized due to other reasons in 9% of patients. Data on use of OTC drugs were not gathered prior to the baseline visit.

A total of 54% and 33% of patients, respectively, had a history of dyspeptic symptoms and reflux (erosive) esophagitis at baseline. Mean duration of GERD symptoms was 7.8 years. Some 76% of patients had undergone upper gastrointestinal endoscopy within 6 months prior to baseline, with findings reported in 74% of patients. Baseline clinical characteristics are presented in Table 1.

**Table 1:** Clinical characteristics from 6 months prior to baseline to baseline visit

**Variable**	**n=262 (%)**
**Gastrointestinal history**	
Dyspeptic symptoms	141 (54)
Hiatal hernia	103 (39)
Reflux esophagitis	86 (33)
Endoscopic findings*	
Hiatal hernia	84 (32)
Reflux esophagitis	50 (19)
Barrett’s esophagus	16 (6)
Esophageal stricture	3 (1)
Duodenal ulcer	2 (0.8)
Other abnormal esophageal findings	28 (11)

* 11.5% of patients underwent upper gastrointestinal endoscopy with biopsy and 21% underwent the procedure without biopsy; an additional 44% of patients had the procedure without further details specified.

### GERD-related resource utilization

Use of prescribed GERD medication generally remained constant over the 12-month study period. Esomeprazole was the most commonly prescribed GERD medication, with use reported in 46% of patients between baseline and 12 months (Table 2). Between baseline and 6 months, the most common test or procedure was endoscopy, reported in 5% of patients, while the most common procedures between 6 and 12 months were blood sampling for laboratory tests (6% of patients) and endoscopy (4%). Over the 12-month study period, primary care physician visits were reported for 32% and 74% of patients on CRF forms and patient surveys, respectively, while gastroenterologist visits were reported for 19% and 35% of patients, respectively. A total of 6 patients (2%) were reported as being hospitalized during the study.

**Table 2:** Use of GERD medications between baseline and 12 months’ follow-up (n=258)

**Drug**	**n (%)**
Esomeprazole	119 (46)
Pantoprazole	45 (17)
Rabeprazole	39 (15)
Lansoprazole	30 (12)
Omeprazole	21 (8)
Cimetidine	2 (0.8)
Famotidine	1 (0.4)
Misoprostol	1 (0.4)
Sucralfate	1 (0.4)
Other drugs for peptic ulcer and GERD	79 (31)

### Symptom burden, HRQL and productivity impairment

At baseline, GERD symptoms (reported using the RESQ-7, Overall symptoms domain) were reported as moderate-to-severe in 98% of patients. Patients’ most frequent symptom from the Heartburn and Regurgitation domains were frequently reported as moderate-to-severe in intensity (95% and 79%, respectively), while patients’ most frequent symptoms from the Burping and Hoarseness, cough and difficulty swallowing domains were rated as moderate-to-severe in 53% and 35% of patients, respectively. A total of 48% of patients reported daily symptoms of GERD despite optimized PPI therapy. The frequency and severity of GERD symptoms appeared to decline over the 12-month course of the study (data not shown).

**Figure 2:**
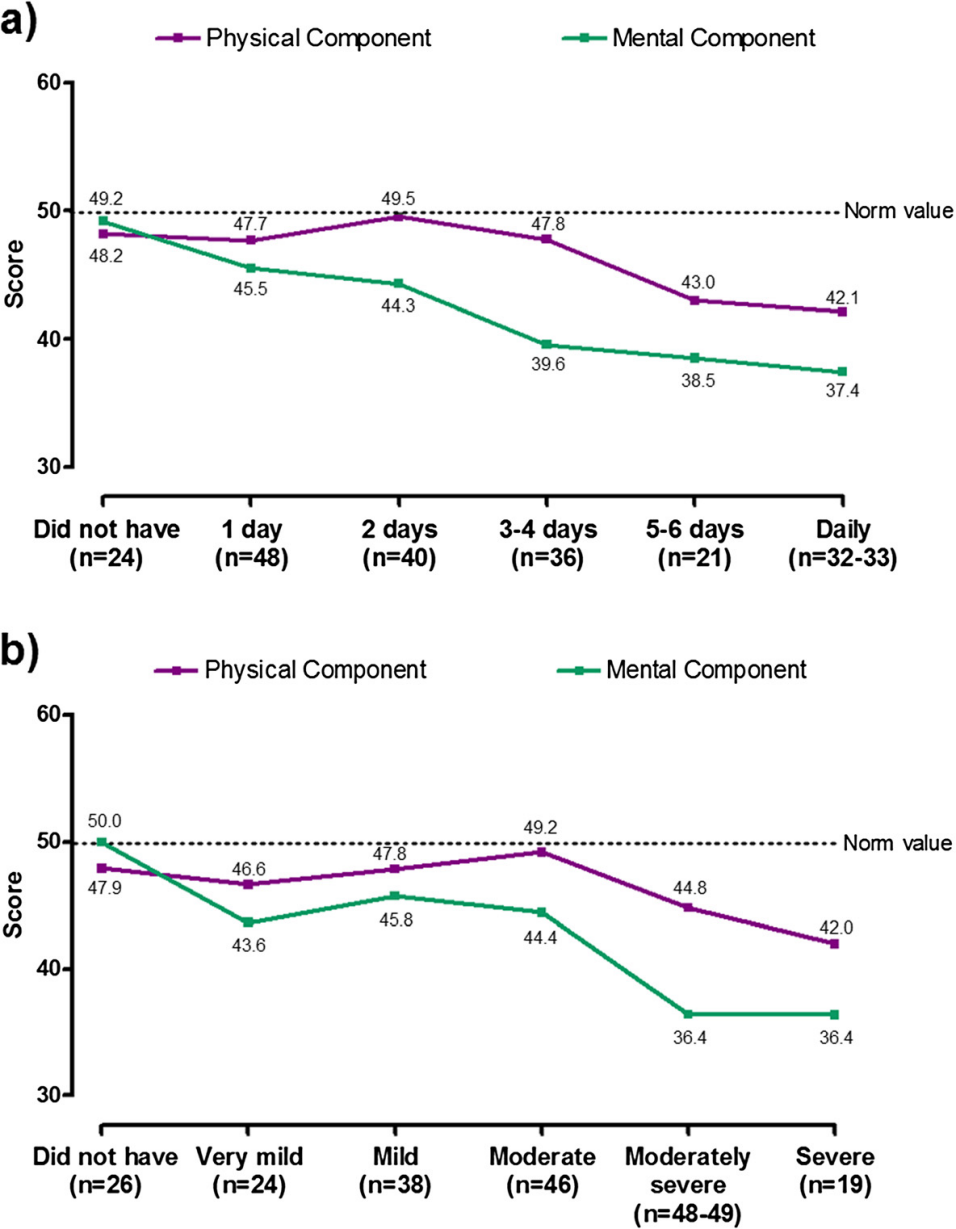
**Mean SF-36v2 Physical and Mental Component summary scores by RESQ-7 Heartburn domain frequency (a) and severity (b) at 6 months’ follow-up.** For both scores, 0 represents worse and 100 represents best health status. Norm values are means derived from the 1998 SF-36 Health Survey of the US general population. Frequency and severity of symptoms were based on the item within the domain with the highest frequency. *The relationship between symptoms and SF-36v2 results is described at the 6-month assessment, since the number of patients in each symptom category was most evenly distributed at this time point.*

**Figure 3:**
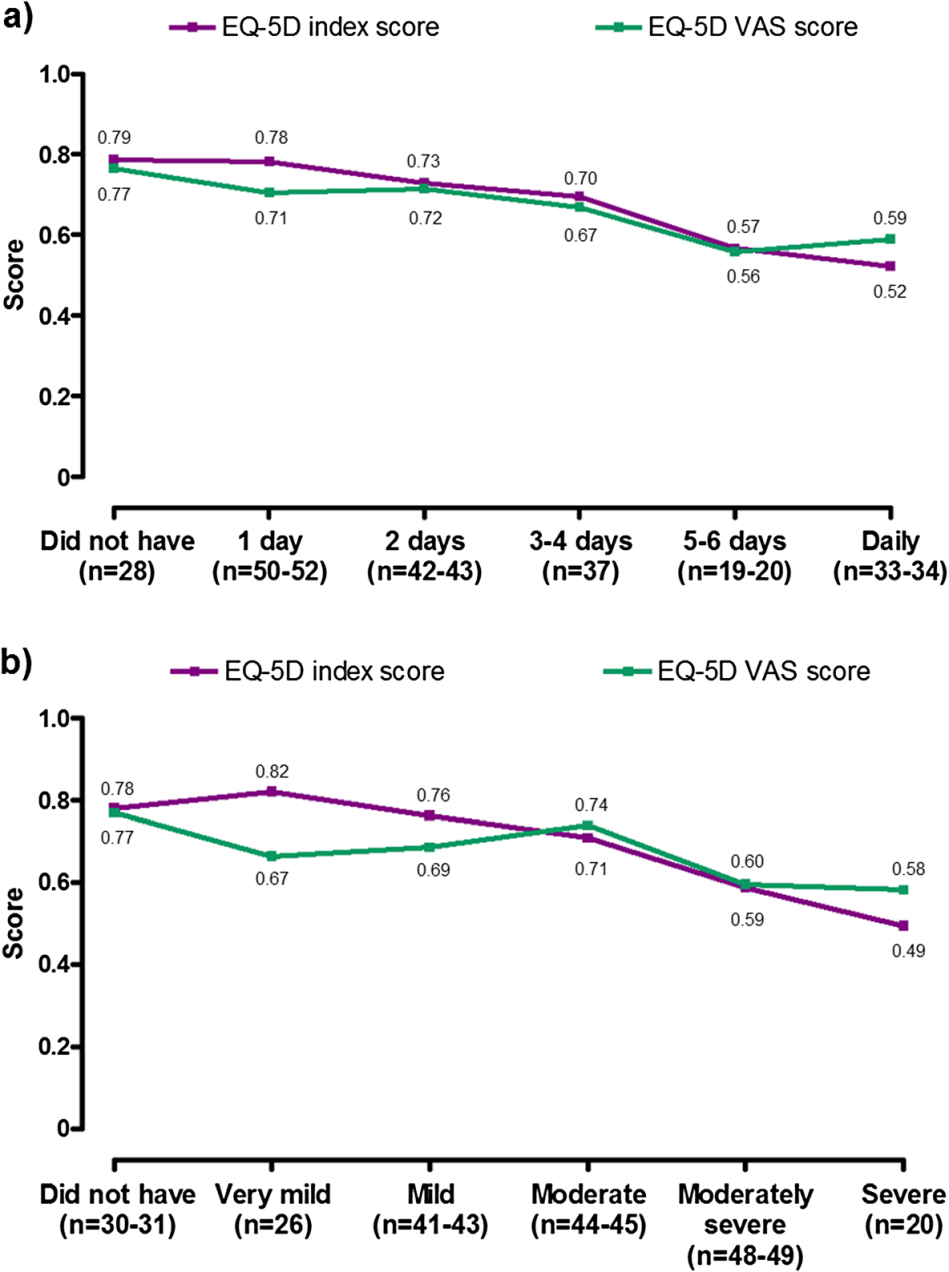
**Mean EQ-5D index and VAS scores by RESQ-7 Heartburn domain frequency (a) and severity (b) at 6 months’ follow-up.** For both EQ-5D scores, 0 represents a health state of being dead and 1 a health state of being at full health (VAS scores were re-scaled from 0–100 to 0–1 for purposes of comparison). Frequency and severity of symptoms were based on the item within the domain with the highest frequency. *The relationship between symptoms and EQ-5D results is described at the 6-month assessment, since the number of patients in each symptom category was most evenly distributed at this time point.*

SF-36v2 and EQ-5D scores at baseline and at 6 and 12 months’ follow-up are shown in Table 3. SF-36v2 Physical and Mental Component summary scores, EQ-5D index score and EQ-5D VAS score all showed a tendency towards lower scores (worse HRQL) with a higher frequency and intensity of GERD symptoms (Figures 2 and 3).

**Table 3:** Mean (95% CI) SF-36v2 Physical and Mental Component summary scores and EQ-5D index and VAS scores at baseline and at 6 and 12 months’ follow-up

	**Baseline**	**6 months**	**12 months**
	**(n=262)**	**(n=260)**	**(n=258)**
**SF-36v2**			
Physical Component	45.0 (43.9, 46.0)	46.7 (45.6, 47.9)	46.9 (45.7, 48.1)
	n=231	n=202	n=210
Mental Component	41.1 (39.6, 42.6)	42.6 (41.1, 44.2)	42.6 (41.0, 44.2)
	n=231	n=201	n=211
**EQ-5D**			
Index score	0.68 (0.65, 0.71)	0.70 (0.66, 0.73)	0.72 (0.69, 0.75)
	n=236	n=212	n=212
VAS*	0.64 (0.61, 0.66)	0.68 (0.65, 0.70)	0.67 (0.65, 0.70)
	n=234	n=211	n=215

* Scores were re-scaled from 0–100 to 0–1 for purposes of comparison.

WPAI-GERD results at baseline showed that the mean time absent from work in the previous 7 days due to GERD was 2.9 hours (95% CI: 1.18, 4.56; n=99) per employed patient, and mean productivity loss while at work was 23.4% (95% CI: 19.2%, 27.5%), corresponding to an average of 7.7 hours (95% CI: 6.2, 9.1; n=101) of work lost due to reduced productivity. Thus, the overall productivity loss was estimated as 10.1 hours (95% CI: 7.9, 12.3) per employed patient. Mean percentage reduction in productivity while carrying out daily activities (for employed and non-working patients) was 34.4% (95% CI: 31.3%, 37.5%; n=236). All WPAI-GERD domain scores appeared to worsen as the frequency and severity of GERD symptoms increased (data not shown).

### Treatment pathways

Over time, the most common treatment pathway was that patients remained on PPI monotherapy. At month 12 of follow-up (n=258), around one third of patients (33.7%) were receiving PPI monotherapy and a small proportion (8.5%) were receiving adjunctive agents (most commonly alginates or domperidone) or other medication (0.8%). The remainder were receiving no medication at this timepoint. Notably, the proportion of patients receiving no medical therapy increased over time (from 20.2% of patients during months 1–6 of follow-up to 57.0% at month 12).

### Costs

In the 6 months prior to baseline, the mean total cost per patient was €3726, of which €361 (10%) was direct costs and €3365 (90%) was indirect costs due to absence from work and reduced productivity. Over the 12-month follow-up period, the mean total cost per patient was €5237, of which €563 (11%) was direct costs and €4674 (89%) was indirect costs. Total costs per patient are presented in Table 4.

**Table 4:** Mean (95% CI) total costs per patient for 6 months prior to baseline and months 1–12

**Cost; € (95% CI)**	**6 months prior to baseline (n=262)**	**Months 1–12 (n=183)**
Total costs	3726 (2876, 4575)	5237 (3949, 6526)
Total direct costs*	361 (327, 394)	563 (503, 623)
Primary care visits**		99 (86, 112)
Specialist visits**		45 (36, 54)
Emergency room visits**		0.14 (-0.13, 0.41)
Hospitalisations**		25 (-4, 55)
Medication	203 (184, 222)	351 (314, 389)
**Tests and procedures**	157 (129, 186)	42 (20, 64)
Total work productivity loss cost	3365 (2515, 4216)	4674 (3385, 5964)
Cost of absence from work	906 (360, 1452)	799 (232, 1366)
Cost of reduced productivity at work	2460 (1860, 3060)	3875 (2854, 4897)

* Total direct costs include only costs of tests and procedures and costs of GERD-related prescribed medications; ** This information was not collected in the 6-month period prior to baseline.

## Discussion

This non-interventional, observation study shows that there is a high symptom load and substantial impairment of HRQL and work productivity among French patients with persistent symptoms of GERD despite optimized PPI therapy, with 98% of patients reporting moderate-to-severe symptoms and 48% reporting daily symptoms at baseline. This symptom burden was consistent with the study inclusion criteria of patients with GERD symptoms at least 3 days/week despite optimized PPI therapy.

The symptom burden in the study population appeared to decrease over the 12-month course of the study. This was expected, as the patients had a high symptom load at recruitment and GERD symptoms tend to fluctuate over time [17,18]. Another possible explanation is increased patient compliance with prescribed PPI therapy as a result of being enrolled in this study. It is important to note, however, that the REMAIN France study was not designed to evaluate changes in symptoms over time (or GERD treatment compliance), and therefore comparisons between baseline and follow-up should be interpreted with caution.

In addition to the high symptom load, the study population was found to have impaired HRQL as assessed by the EQ-5D and SF-36v2 scales, with the level of impairment being higher in patient categories with more frequent and/or severe symptoms. This finding was consistent with observations from several studies that assessed HRQL in patients with GERD using the EQ-5D and SF-36 instruments. For example, Kartman et al. [19] surveyed 1011 patients with GERD in Sweden and Germany, assessing HRQL on the EQ-5D, as well as the frequency and severity of heartburn. The study found that patients with heartburn assign themselves an impaired utility value, and that there was a correlation between utility impairment and the severity and frequency of their symptoms.

A number of studies have assessed the impact of GERD on HRQL using the SF-36v1 [20-25], with all studies showing a significant impairment of quality of life in patients with GERD compared with the general population, both on the Physical and Mental Component summary scores and the individual domains of the SF-36v1. As with the EQ-5D, the degree of impairment of HRQL as assessed on the SF-36v1 was related to symptom load, with HRQL being impaired to a greater degree in patients with a greater symptom burden [20-26]. The findings of the present study are therefore in agreement with the latter observations and the results of a US study [5], which found similar impairment of HRQL on the EQ-5D and SF-36v2 scales in North American patients with a partial response to optimized PPI therapy, as well as a similar relationship between the severity and frequency of symptoms and the level of impairment of HRQL.

This study population showed evidence of impaired work productivity on the WPAI-GERD scale, with increased absenteeism and presenteeism resulting in considerable indirect costs. Impaired work-related productivity has been observed in patients with GERD in other studies [26-28], with the studies by Gisbert et al. and Ekesbo et al. both being examples of studies assessing productivity loss using the WPAI-GERD scale in a European population. Both HRQL and impairment of productivity was shown to be higher in those patients with greater GERD symptom load in the present study, results that are consistent with those of the REMAIN US study [5].

The relationships between symptom load, measured by the validated RESQ-7, and the WPAI-GERD and EQ-5D questionnaires lends indirect support to the construct validity of these instruments in this study population, as the severity and frequency of GERD symptoms measured by the RESQ-7 appears to have some predictive value for the degree of impairment of HRQL and work-related productivity. However, it should be noted that this study was not designed to test the construct validity of these scales.

Though the current patient population continued to experience frequent and severe symptoms of GERD despite receiving optimized treatment with a PPI with or without adjunctive therapy with other GERD treatments, only a low proportion of patients were observed to change treatment categories over the course of the study. Based on these data, it appears that there is currently no clear treatment alternative to continuing PPI therapy in patients with partially responsive GERD. While the possibility of incorrect diagnosis has to be considered, it is important to note that participating patients had been previously diagnosed with GERD (at least 6 months previously, according to medical records) and all had been receiving optimized PPI therapy according to physician’s judgement. With regard to GERD diagnosis, we adopted the specified timeframe of patients having been diagnosed >6 months beforehand in order to help to confirm the diagnosis in terms of duration. Although imperfect, this duration constraint helped to avoid inclusion of patients with a less well established diagnosis or very intermittent forms of GERD. In addition, two thirds of patients had endoscopically proven GERD, which is a higher rate than would usually be expected for such patients, and symptoms were confirmed by use of a validated questionnaire (RESQ-7).

This study has a number of strengths, including the large sample size and the use of established patient-reported outcome measures to evaluate HRQL and productivity. Use of a pragmatic definition of partial response – persistent GERD symptoms despite optimized PPI therapy, according to the physician’s judgement – can also be considered a study strength as it helped to ensure that the study mirrored routine practice. In this regard it is important to consider that numerous guidelines have attempted to define partial response. However, we deliberately avoided proving physicians with clinical training on the management of GERD or related guidelines, in order to not interfere with their routine management of patients with GERD and, in turn, to obtain real-world data. Factors limiting the study include the use of patient recall periods of up to 3 months when recording resource utilization, although the potential impact of memory decay was partly overcome by providing patients with memory aids (diaries) in which they were encouraged to document use of medical care and medications for GERD between physician visits. The measurement of symptoms using a 7-day recall rather than daily measurement may also be a study limitation, as well as the substantial amount of missing data such as medication dosages and treatment dates. In addition, the discrepancy between the number of office visits recorded by physicians compared with the number reported by patients highlights the limitations of collecting data from medical records and patient recall. The methods used to calculate costs were also subject to several limitations, including the effects of the unit costs on cost-of-illness calculations (which may be subject to variability), the extrapolation of WPAI-GERD data over 7 days to a 3- to 6-month period to estimate productivity losses and the subjective method of measuring work productivity.

## Conclusions

There is a high symptom load (in terms of frequency and/or severity) and, in turn, substantial impairment of HRQL and work productivity and high disease-related costs among patients in France with persistent symptoms of GERD despite PPI therapy. Such findings highlight the requirement for new treatment options to meet the unmet need of such patients. In the meantime, physicians are encouraged to explore not only the possibility that PPI treatment is not optimized (even if they believe it to be) but also the likelihood of incorrect diagnosis and/or the potential benefits of adjunctive therapies.

## Abbreviations

GERD: Gastrointestinal reflux disease; HRQL: Health-related quality of life; PPI: Proton pump inhibitor; HADS: Hospital Anxiety and Depression Scale; OTC: Over the counter; CRF: Case report forms.

## Competing interests

SBV is a speaker, consultant and/or advisory board member for AstraZeneca, Cephalon, Danone Research, Given Imaging, Iprad, Ipsen Beaufour, Janssen-Cilag, Novartis, Nycomed and Takeda, and has received research funding from AstraZeneca, Given Imaging, Janssen-Cilag and Shire Pharmaceuticals. HGL, MK, PW, MR and N-OS are current (or former) employees of AstraZeneca. MLF and ND are current (or former) employees of OptumInsight, who conducted the study with funding from AstraZeneca.

## Authors’ contributions

Study planning and interpretation of data: SBV Study planning, monitoring of conduct and interpretation of data: HGL, PW and N-OS Monitoring of study conduct and interpretation of data: MK Study planning and interpretation of data: MR Study planning, conduct and interpretation of data: MLF and ND All authors contributed to drafting and critical revision of the manuscript, and approved the final version for submission for publication.

## Pre-publication history

The pre-publication history for this paper can be accessed here:

http://www.biomedcentral.com/1471-230X/13/39/prepub

## References

[B1] Dent J El-Serag HB Wallander MA Johansson S Epidemiology of gastro-oesophageal reflux disease: a systematic review Gut 2005 54 5 710 717 10.1136/gut.2004.051821 15831922 PMC1774487

[B2] Bruley des Varannes S Marek L Humeau B Lecasble M Colin R Gastroesophageal reflux disease in primary care. Prevalence, epidemiology and quality of life of patients Gastroenterol Clin Biol 2006 30 3 364 370 10.1016/S0399-8320(06)73189-X 16633300

[B3] Kahrilas PJ Shaheen NJ Vaezi MF American Gastroenterological Association Institute technical review on the management of gastroesophageal reflux disease Gastroenterology 2008 135 4 1392 1413 10.1053/j.gastro.2008.08.044 18801365

[B4] El-Serag H Becher A Jones R Systematic review: persistent reflux symptoms on proton pump inhibitor therapy in primary care and community studies Aliment Pharmacol Ther 2010 32 6 720 737 10.1111/j.1365-2036.2010.04406.x 20662774

[B5] Stålhammar N-O Spiegel BM Granstedt Löfman H Karlsson M Wahlqvist P Næsdal J Nelson MT Despiégel N Partial response to proton pump inhibitor therapy for GERD: observational study of patient characteristics, burden of disease, and costs in the USA Pragmatic Observ Res 2012 3 57 67 10.2147/POR.S36704 PMC5045010 27774018

[B6] Vakil N Karlsson M Denison H Rydén A A patient reported outcome instrument in partial responders to proton pump inhibitor therapy suggests new symptoms deserve consideration: results from a validation study Gut 2011 60 Suppl. 3 A266

[B7] Shaw MJ Talley NJ Beebe TJ Rockwood T Carlsson R Adlis S Fendrick AM Jones R Dent J Bytzer P Initial validation of a diagnostic questionnaire for gastroesophageal reflux disease Am J Gastroenterol 2001 96 1 52 57 10.1111/j.1572-0241.2001.03451.x 11197287

[B8] Snaith RP The hospital anxiety and depression scale Health Qual Life Outcomes 2003 1 29 10.1186/1477-7525-1-29 12914662 PMC183845

[B9] Ware JE Jr Sherbourne CD The MOS 36-item short-form health survey (SF-36). I. Conceptual framework and item selection Med Care 1992 30 6 473 483 10.1097/00005650-199206000-00002 1593914

[B10] Rabin R de Charro F EQ-5D: a measure of health status from the EuroQol Group Ann Med 2001 33 5 337 343 10.3109/07853890109002087 11491192

[B11] Wahlqvist P Medin J Karlsson M Reilly MC Responsiveness to change and construct validity of the Work Productivity and Activity Impairment questionnaire for gastroesophageal reflux disease (WPAI:GERD) in Swedish patients Value Health 2009 12 3 A60

[B12] Base des médicaments et informations tarifaires http://www.codage.ext.cnamts.fr/codif/bdm_it/index.php?p_site=AMELI Accessed 25 March 2013

[B13] Tarifs conventionnelshttp://www.ameli.fr/professionnels-de-sante/medecins/votre-convention/tarifs/tarifs-conventionnels-des-medecins-generalistes/tarifs-des-medecins-generalistes-en-metropole.php Accessed 25 March 2013

[B14] Consommation en santé\Activité des professions de santé libérales\Professions médicales\Généralistes libéraux\Dépassements\Valeur moyenne du dépassement http://www.ecosante.org/index2.php?base=FRAN&langh=FRA&langs=FRA&sessionid=) Accessed 25 March 2013.

[B15] Démographie et honoraires des médecins libéraux en 2006 http://www.ameli.fr/fileadmin/user_upload/documents/DP_honoraires_2006_def.pdf Accessed 25 March 2013.

[B16] Coût mensuel de la main d’oeuvre (tps00174) http://epp.eurostat.ec.europa.eu/portal/page/portal/labour_market/labour_costs/main_tables Accessed 25 March 2013.

[B17] Isolauri J Luostarinen M Isolauri E Reinikainen P Viljakka M Keyrilainen O Natural course of gastroesophageal reflux disease: 17-22 year follow-up of 60 patients Am J Gastroenterol 1997 92 1 37 41 8995934

[B18] Malfertheiner P Nocon M Vieth M Stolte M Jaspersen D Koelz HR Labenz J Leodolter A Lind T Richter K Evolution of gastro-oesophageal reflux disease over 5 years under routine medical care–the ProGERD study Aliment Pharmacol Ther 2012 35 1 154 164 10.1111/j.1365-2036.2011.04901.x 22070159

[B19] Kartman B Gatz G Johannesson M Health state utilities in gastroesophageal reflux disease patients with heartburn: a study in Germany and Sweden Med Decis Making 2004 24 1 40 52 10.1177/027298X03261563 15005953

[B20] Revicki DA Wood M Maton PN Sorensen S The impact of gastroesophageal reflux disease on health-related quality of life Am J Med 1998 104 3 252 258 10.1016/S0002-9343(97)00354-9 9552088

[B21] Wahlqvist P Symptoms of gastroesophageal reflux disease, perceived productivity, and health-related quality of life Am J Gastroenterol 2001 96 8 Suppl S57 S61 11510773 10.1016/s0002-9270(01)02590-4

[B22] Kulig M Leodolter A Vieth M Schulte E Jaspersen D Labenz J Lind T Meyer-Sabellek W Malfertheiner P Stolte M Quality of life in relation to symptoms in patients with gastro-oesophageal reflux disease– an analysis based on the ProGERD initiative Aliment Pharmacol Ther 2003 18 8 767 776 10.1046/j.1365-2036.2003.01770.x 14535869

[B23] El-Dika S Guyatt GH Armstrong D Degl’innocenti A Wiklund I Fallone CA Tanser L Veldhuyzen van Zanten S Heels-Ansdell D Wahlqvist P The impact of illness in patients with moderate to severe gastro-esophageal reflux disease BMC Gastroenterol 2005 5 23 10.1186/1471-230X-5-23 16004616 PMC1183201

[B24] Pacini F Calabrese C Cipolletta L Valva MD Russo A Savarino V Vigneri S Burden of illness in Italian patients with gastro-oesophageal reflux disease Curr Med Res Opin 2005 21 4 495 502 10.1185/030079905X38231 15899097

[B25] Ronkainen J Aro P Storskrubb T Lind T Bolling-Sternevald E Junghard O Talley NJ Agreus L Gastro-oesophageal reflux symptoms and health-related quality of life in the adult general population–the Kalixanda study Aliment Pharmacol Ther 2006 23 12 1725 1733 10.1111/j.1365-2036.2006.02952.x 16817916

[B26] Ekesbo R Sjöstedt S Sörngård H Effects of structured follow-up and of more effective acid inhibitory treatment in the management of GORD patients in a Swedish primary-care setting: a randomized, open-label study Clin Drug Investig 2011 31 3 181 189 10.2165/11586330-000000000-00000 21288053

[B27] Gisbert JP Cooper A Karagiannis D Hatlebakk J Agreus L Jablonowski H Nuevo J Impact of gastroesophageal reflux disease on work absenteeism, presenteeism and productivity in daily life: a European observational study Health Qual Life Outcomes 2009 7 90 10.1186/1477-7525-7-90 19835583 PMC2770561

[B28] Liker H Jones R Ducrotté P The effect of sleep disturbance due to gastroesophageal reflux disease on work and leisure productivity: results from a multinational survey Gastroenterology 2005 128 4 Suppl 2 A386

